# Relationships Between Stigma and Intimate Partner Violence Among Female Sex Workers Living With HIV: Social and Economic Exclusion

**DOI:** 10.1177/10778012221127722

**Published:** 2022-11-07

**Authors:** Amelia Rock, H. Luz McNaughton Reyes, Vivian Go, Suzanne Maman, Martha Perez, Yeycy Donastorg, Deanna Kerrigan, Clare Barrington

**Affiliations:** 1 Department of Health Behavior, UNC Gillings School of Global Public Health, USA; 2 HIV Vaccine Research Unit, Instituto Dermatológico y Cirurgia de Piel Dr. Humberto Bogaert Diaz, Dominican Republic; 3 Department of Prevention and Community Health, GWU Milken Institute School of Public Health, USA

**Keywords:** Dominican Republic, stigma, intimate partner violence, sex work, HIV

## Abstract

Violence against female sex workers (FSWs) perpetrated by their intimate (i.e., non-commercial) partners, particularly against FSWs living with HIV, is understudied. Stigma can deplete the economic resources, social relationships, and mental well-being of stigmatized people, which may increase their intimate partner violence (IPV) risk. We quantitatively assessed relationships between HIV stigma and sex work stigma and IPV victimization among FSWs living with HIV in the Dominican Republic (*n* = 266). Enacted HIV stigma, in the form of job loss, and anticipated HIV stigma, in the form of fear of exclusion by family, were associated with increased IPV risk. Potential association mechanisms, including increased economic vulnerability and social isolation, and programmatic responses are discussed.

## Background

### Intimate Partner Violence Against Female Sex Workers, Stigma, and Research Limitations

Violence victimization is associated with a host of adverse physical and mental health outcomes among women, including female sex workers (FSWs), such as suboptimal HIV treatment effectiveness (Bacchus et al., 2018; Campbell, 2002; Dasgupta, 2021; Decker et al., 2015; Hatcher et al., 2015; Lyons et al., 2017; Mendoza et al., 2017; Shannon et al., 2015; Ulibarri et al., 2013). Less empirical research, policy, and programming has been dedicated to preventing violence against FSWs—women who exchange sexual acts for money or goods—than to violence against women generally, in part a reflection of their historical exclusion from global organizing around violence against women (Cabezas, 2009). Over the past decade, international agendas on violence prevention and research on violence risk factors have increasingly included FSWs (Argento et al., 2021; Deering et al., 2014). However, research on risk factors for intimate partner violence (IPV)—violence perpetrated against FSWs by their intimate partners, that is, boyfriends, husbands, and casual sex partners who do not pay for sex acts—remains limited (Argento et al., 2021; Deering et al., 2014). The literature on IPV against FSWs living with HIV is especially small.

Studies have found that correlates of violence against FSWs differ by perpetrator, for example, clients and intimate partners (Decker et al., 2020; Hail-Jares et al., 2015). This suggests that intimate partner-perpetrated violence, IPV, has a unique web of causal factors to be understood. However, studies of risk factors for violence against FSWs frequently do not specify or disaggregate results by perpetrator (e.g., Ngale et al., 2019; Reed et al., 2011; Shannon et al., 2009). Among etiological studies of violence against FSWs that do specify perpetrator, the majority examines violence perpetrated by clients, police, or others related to their work (Argento et al., 2021; Deering et al., 2014), even though, in many settings, violence perpetrated by their intimate partners appears more common (e.g., Cowan et al., 2017; Hong et al., 2013; Mendoza et al., 2017). A lack of comprehensive understanding of IPV risk factors may hamper the design of effective IPV prevention interventions for FSWs, which, to date, have been few (Deering et al., 2014; Javalkar, Platt, Prakash, Beattie, Bhattacharjee, et al., 2019).

The existing literature on IPV risk factors among FSWs has identified relationship control (Luchters et al., 2013; Ulibarri et al., 2014), partner jealousy (Decker et al., 2013), FSWs’ financial support of partners (Argento et al., 2014), mental health (Hong et al., 2013), substance use (Chersich et al., 2014), client violence (Javalkar, Platt, Prakash, Beattie, Collumbien, et al., 2019), social isolation (Hail-Jares et al., 2015), entry to sex work through force (Decker et al., 2020), and homelessness (Decker et al., 2020). Few studies of FSWs have examined the influence of structural risk factors—contextual factors at the macrostructural and community levels (Parkhurst, 2014; Shannon et al., 2014 2015; STRIVE Research Consortium, 2019)—such as stigma, on IPV risk.

FSWs living with HIV contend with both HIV-related stigma and sex work-related stigma, which are co-constitutive and mutually exacerbating (Logie et al., 2011; Parker & Aggleton, 2003; Pheterson, 1993). Per Link and Phelan (2001), stigma is the co-occurrence of five processes: labeling of people who have a socially significant characteristic, linking of labels to negative meanings and stereotypes, and separation of, discrimination against, and social status loss of the stigmatized. FSWs and people living with HIV (PLHIV) have themselves identified stigma as a pervasive and powerful threat to their health and well-being, and empirical studies indicate its adverse effects on myriad health and social outcomes (e.g., Red de Trabajadores Sexuales de Latinoamérica y el Caribe [RedTraSex], 2007; Rueda et al., 2016; Shannon et al., 2018).

Stigma may also contribute to the relative paucity of research on IPV risk factors among FSWs. Stigma-rooted stereotypes of sex work as inherently, universally violent and FSWs as victims of this violence—which do not reflect the context-dependent empirical variability in the burden of violence FSWs experience while working, or in their reported subjective experiences of sex work (Bernstein, 1999; Sanders, 2016; Weitzer, 2009)—may contribute to the disproportionate focus on violence against FSWs that is work-related. The dehumanizing stereotype that FSWs do not have intimate partners (Strathdee et al., 2015) may also contribute to the lack of research on intimate partner-perpetrated violence among FSWs.

The disproportionate focus in research on FSWs’ working lives obscures their full identities and experiences. As Deborah Brock (1998, p. 11) observed, “women working in prostitution *become* prostitutes in the eyes of others; that is, publicly they are more identified with their work than are people in other jobs.” This reduction of individuals to a stigmatized social characteristic reflects the stigmatization process of separating the stigmatized—“them,” the other—from the dominant group, “us” (Link & Phelan, 2001). Thus, the relative paucity of research on IPV among FSWs may both reflect and reproduce sex work stigma.

### Stigma and Pathways of Influence on IPV among FSWs Living With HIV

To expand the limited literature on IPV risk factors among FSWs and FSWs living with HIV, and specifically on stigma as an IPV risk factor, we quantitatively assessed relationships between stigma related to HIV and sex work and IPV victimization among FSWs living with HIV. Per fundamental cause theory of stigma, stigma produces negative health outcomes among stigmatized people through multiple pathways, including reduction of their available resources (e.g., money, health care, and law enforcement protection), undermining of their social relationships, and spurring of harmful psychological and behavioral responses (e.g., substance use and depression) (Hatzenbuehler et al., 2013). This theory, when triangulated with research on IPV risk factors, indicates how stigma may lead to increased IPV risk among FSWs living with HIV, as detailed below.

Regarding the pathway linking stigma and IPV through reduction of available resources, FSWs living with HIV, who often work both in and outside the sex industry, report enacted stigma in multiple work environments that depletes economic resources (Human Rights Watch, 2004; Rael et al., 2016). For example, involuntary HIV testing in factories and hospitality industry jobs leads to loss of employment, and status outing by colleagues in sex work establishments leads to client and income loss (Barrington et al., 2017; Human Rights Watch, 2004; Kennedy et al., 2013; Rael et al., 2016; Zulliger et al., 2017). Law enforcement discrimination against FSWs, such as random arrest and extortion, also creates economic costs (Brennan, 2004; Gregory, 2007). Depleted economic resources—such as employment and income, which serve as intimate partner relationship “exit options” (Caridad Bueno & Henderson, 2017, p. 5)—may, in turn, reduce women's power within and/or ability to leave intimate partner relationships, which may increase their risk of IPV, per feminist economic household bargaining models (Deere & Doss, 2006; Friedemann-Sánchez, 2006; Safa, 1995).

Supporting the pathway linking stigma and IPV via undermining of social relationships, studies show that enacted stigma in the form of exclusion and abuse of stigmatized people by their social network members, and anticipated such stigmatization, may decrease access to emotional and financial social support, critical resources for contending with and escaping IPV (Go et al., 2011; Hail-Jares et al., 2015; Sandelowski et al., 2004). Finally, regarding the pathway linking stigma and IPV via adverse psychological and behavioral responses to stigma, internalized, anticipated, and enacted stigma can lead to depression, diminished self-worth, and substance use, which can hamper escape from abuse, and are associated with heightened IPV victimization risk (Bacchus et al., 2018; Chen et al., 2015; Hong et al., 2013; Li et al., 2010; Rock et al., 2017; Witte et al., 2010).

We tested the hypotheses that HIV stigma and sex work stigma would be associated with increased odds of IPV. Given the empirical findings that different “mechanisms” of stigma—that is, enacted, anticipated, and internalized stigma—relate to different health outcomes (Earnshaw, Bogart, et al., 2013), and to the same outcomes through differing pathways (Sweeney & Vanable, 2016), we examined relationships between each of these stigma mechanisms and IPV. Findings may be useful for the design of programs and policies to reduce the burden of IPV by intervening to address particular mechanisms and forms of stigma.

## Methods

### Study Setting and Parent Study

We analyzed baseline survey data from an evaluation of *Abriendo Puertas* (Opening Doors), an intervention promoting HIV care and prevention among a cohort of FSWs living with HIV in Santo Domingo, DR (2012–2014; Kerrigan et al., 2016). HIV prevalence among FSWs in the DR is approximately 4.4% (Consejo Nacional para el VIH y el SIDA, 2012), five-fold greater than the national adult HIV prevalence of 0.9% (UNAIDS, 2021). Mendoza et al. (2017) found that 18.3% of the *Abriendo Puertas* cohort at baseline reported violence from a sexual partner in the last 6 months, with a greater proportion reporting violence from intimate partners (12.3%) than from clients (8.3%), and some (2.6%) reporting both. Among women generally in the DR, IPV is the fourth leading cause of death (Centro de Estudios de Género & Instituto Tecnológico de Santo Domingo, 2013). Anthropological and public health research illustrate anti-violence against women discourse in public space and media coverage, popular awareness of the issue, and seemingly low social acceptability: the percentage of Dominican people agreeing to at least one instance where “wife-beating” is justified is low (6%), particularly when compared to other low- and middle-income countries (Cabezas, 2009; Caridad Bueno, 2013; United Nations General Assembly, 2000). However, the landscape of norms and attitudes toward IPV is multifaceted and contradictory—qualitative studies suggest that victim blaming and normalization of IPV are pervasive (Breitbart et al., 2010; Viswanathan et al., 2016). Selling sexual services is not illegal in the DR, but it is illegal to profit from earnings of sex workers and facilitate prostitution (Cabezas, 1999). In some areas, FSWs are commonly treated as criminals by law enforcement, for example, randomly arrested (Amnesty International, 2019; Cabezas, 2004; Gregory, 2007).

Participants were recruited via non-random sampling led by peer navigators (current and former FSWs experienced in conducting HIV prevention and support; Donastorg et al., 2014). Study inclusion criteria included being 18 years of age or older, HIV-positive, and exchanging sex for money in the last month. HIV status was confirmed via a single rapid test (Retrocheck) prior to the baseline survey (Donastorg et al., 2014). From November 2012 to February 2013, 268 FSWs were enrolled and completed a baseline survey. A trained Dominican female interviewer administered structured paper surveys to participants in Spanish in a private office. Prior to data collection, participants provided consent orally to protect confidentiality. Study protocols and consent procedures were approved by the Institutional Review Boards of the Johns Hopkins Bloomberg School of Public Health, the University of North Carolina, and the Instituto Dermatologico y Cirugia de Piel Dr. Humberto Bogart Diaz, the Dominican research partner for the study, which oversaw all local data collection. Participants received approximately 10 U.S. dollars for completing each survey visit.

## Measures

### Dependent Variable

***Intimate partner violence***. Seven questions, adapted from the WHO Violence Against Women Instrument (Schraiber et al., 2010), asked whether or not participants had experienced particular acts of physical and sexual violence (e.g., been pushed, kicked, and forced to have sex) in the last 6 months. The questions were asked separately regarding intimate partners, new clients, and regular clients. Intimate partners were defined as sexual partners with whom a participant had sex three or more times and who did not pay per sexual act although they may have given her money. If a participant answered “yes” to any of the questions regarding violence perpetrated by an intimate partner, she was considered to have experienced IPV. The IPV questions were posed to participants who replied affirmatively to one or more preceding survey questions regarding experiences of conflict with or maltreatment from sexual partners.

### Independent Variables

Survey items separately measuring HIV stigma and sex work stigma were adapted from existing HIV stigma scales (Berger et al., 2001; Zelaya et al., 2008) and are based on the Earnshaw, Smith, et al. (2013) HIV Stigma Framework. These items were used to create separate HIV stigma and sex work stigma variables for statistical models, presented in Tables 1–3.

**Table 1.** Enacted Sex Work and HIV Stigma Measures

Model variables	Survey item(s)
Index mean score range: 0–1	Response options: Ever experienced = 1/else = 0
Social discrimination (HIV, sex work)	Felt excluded from family get-togethers because you exchange sex for money/are living with HIV? Felt that members of your family have made discriminatory comments or have gossiped about you because you exchange sex for money/are living with HIV? Felt rejected by friends because you exchange sex for money/are living with HIV?
Health service discrimination (HIV, sex work)	Felt you have been given bad services in a health center because you exchange sex for money/are living with HIV? Been denied medical care because you exchange sex for money/are living with HIV? Heard health service personnel gossiping about you because you exchange sex for money/are living with HIV?
Workplace discrimination—job loss (HIV, sex work)	Lost a job because you exchange sex for money/are living with HIV?
Law enforcement discrimination (Sex work)	Felt that the police neglected to protect you because you exchange sex for money? Been insulted or threatened by the police because you exchange sex for money? Experienced abuse or attempted physical or sexual abuse by the police because you exchange sex for money? Been imprisoned or detained because you exchange sex for money?

**Table 2.** Anticipated HIV Stigma Measures.

Model variables/survey items
Response options: Strongly agree = 4/Agree = 3/Disagree = 2/Strongly disagree = 1
You are afraid you could be threatened with violence if your HIV status were known
You are afraid that if you disclosed your HIV status to your friends, they would lose respect for you
You are afraid that if you disclose your HIV status to the women with whom you work, they could take your clients
You are afraid your partner could leave you if your HIV status were known
You are afraid that your family could exclude you from regular family activities if your HIV status were known

**Table 3.** Internalized HIV and Sex Work Stigma Measures

Model variables	Survey items
Index mean score range: 1–4	Response options: Strongly agree = 4/Agree = 3/Disagree = 2/ Strongly disagree = 1
Internalized stigma (HIV, sex work)	Sex work/HIV makes you feel like a bad person. You feel like you're not as good as others because you exchange sex for money/are living with HIV. The attitudes of people toward HIV/sex work make you feel worse about yourself. You feel guilty because you exchange sex for money/are living with HIV. You feel ashamed of exchanging sex for money/living with HIV. It is easier to avoid friends than to tell them that you exchange sex for money/are living with HIV. You feel worthless because you exchange sex for money/are living with HIV. You feel that you bring many problems to your family because you exchange sex for money/are living with HIV.

***Enacted stigma*** is the experience of being discriminated, stereotyped, or prejudiced against due to a social characteristic (Earnshaw & Chaudoir, 2009). Based on a review of multidisciplinary literature (Brennan, 2004; Carrasco et al., 2017; Gregory, 2007; Human Rights Watch, 2004; Rael et al., 2016; Zulliger et al., 2017), we identified five forms of enacted HIV stigma and enacted sex work stigma experienced by people living with HIV (PLHIV) and FSWs in the DR: social discrimination (e.g., verbal abuse and rejection by family and friends), workplace discrimination (job loss due to HIV or FSW status), law enforcement discrimination (sex work-related only; e.g., refusal to provide protection to FSWs, sexual abuse, and arbitrary arrest), and health service discrimination (e.g., providers refusing to provide services and poor service quality). To measure these constructs, binary (yes/no) survey items assessing lifetime enacted stigma experiences were combined to create multi-item indices (three to four items each), except for workplace HIV and sex work discrimination (job loss), which were assessed with single binary items as these were the only available items assessing those experiences. Mean scores were calculated for each multi-item index (index range: 0–1).

***Anticipated HIV stigma*** refers to expectations of discrimination, stereotyping, and/or prejudice from others due to HIV (Earnshaw & Chaudoir, 2009). Five survey items with 4-point Likert scale responses assessed participants’ levels of fears of violence, losing friends’ respect, colleagues taking their clients, partner abandonment, and family exclusion. The items were included in models as discrete variables as they were expected to have diverse relationships with IPV given their varying proximity to the IPV outcome, for example, the item “you are afraid you could be threatened with violence if your HIV status were known” may capture perceived risk of violence, a proximal correlate of IPV, and was thus expected to relate more strongly to IPV than other items. The surveys did not include measures of anticipated sex work stigma.

***Internalized stigma*** is defined as the self-application of negative attitudes toward one's stigmatized characteristic (Earnshaw & Chaudoir, 2009). Two eight-item scales separately assessed participants’ levels of internalized HIV stigma and internalized sex work stigma. Participants indicated agreement with statements such as “You feel worthless because you are a sex worker” on a 4-point Likert scale. The scales showed strong internal reliability (Cronbach's alpha = 0.87). Mean scores were calculated for each scale (scale range: 1–4).

***Socio-demographic and behavioral control variables.*** Measures of theoretically plausible causes of both independent and dependent variables were used as controls, including age (years), educational attainment (any years primary/any secondary or tertiary school), civil status (has cohabiting partner/non-cohabiting partner/no partner, including separated, widowed, divorced), and number of sexual partners (intimate partners and clients) per month.

### Analytic Approach

The analytic sample was comprised of baseline survey participants (*n* = 268). Two (0.75% of participants at baseline) were missing data on key covariates and dropped from the study, yielding a final analytic sample of 266. We produced univariate statistics for all variables and unadjusted bivariate associations between IPV and both stigma measures and control variables using logistic regression, and examined correlations between stigma variables. We assessed problematic multicollinearity amongst independent variables based on Pearson correlations (>.8) and variance inflation factors (≥10) and determined that none needed to be removed on this basis (Kutner et al., 2004). The final model included all stigma variables, presented in Tables 1–3, and covariates. Logistic regression analyses were performed using SAS (version 9.4). We used two-tailed tests and a *p*-value less than or equal to .05 as the significance level for all analyses.

## Results

### Sample Characteristics

Table 4 presents sociodemographic and behavioral characteristics of participants (*n* = 266). The percentage of participants who reported experiencing one or more forms of physical or sexual violence perpetrated by an intimate partner in the past 6 months was 12.0% (*n* = 32). The median age was 36 (range: 18–61) and approximately two-thirds had a primary school level of education (any number of years; 64.3%). The majority had a steady partner, 38.7% cohabiting and 42.1% not cohabiting, and the median number of children was three (range: 0–8). Those who worked in the street during the past 3 months comprised 56%, while others worked in sex establishments or independently (44.0%). The median number of sexual partners in the last month was 12 (range: 1–51).

**Table 4.** Sociodemographic and Behavioral Characteristics of Female Sex Workers Living With HIV in Santo Domingo, by Intimate Partner Violence Victimization (*n* = 266)

	IPV victimization, last 6 months^a^	
	Yes 12.0% (*n* = 32)	No 88.0% (*n* = 234)
Characteristic	Median (range; IQR) or percentage (frequency)	
Age (years)	33.5 (18–52; 23–40.5)	36.0 (18–61; 30–42)
Educational attainment		
Primary school (any number years)	75.0% (24)	62.8% (147)
Any secondary or tertiary school (any number years)	25.0% (8)	37.2% (87)
Civil status		
Lives with spouse/steady partner	28.1% (9)	44.0% (103)
Non-cohabitating steady partner	65.6% (21)	35.0% (82)
Single (no steady partner, separated, divorced, widowed)	6.3% (2)	20.9% (49)
Number of children	2.5 (0–6; 1–4)	3.0 (0–8; 2–4)
Worksite		
The street	53.1% (17)	56.4% (132)
Sex establishment or independent	46.9% (15)	43.6% (102)
Number of sexual partners/month	11.5 (4–51; 10–17.5)	12.0 (1–51; 7–18)
HIV services utilization (times received HIV care, past six months)	3.0 (0–10; 0–5)	3.0 (0–12; 2–6)
Alcohol use frequency (past month)		
Frequent (a few times/week, once/week, weekends)	50.0% (16)	41.9% (98)
Infrequent (once/month, rare occasions, once/two weeks)	25.0% (8)	24.8% (58)
Never	25.0% (8)	41.9% (98)

*Note*. IPV = intimate partner violence.

a IPV victimization is any physical or sexual victimization in the past 6 months.

### Levels of Lifetime Stigma Experienced and Bivariate Associations With IPV

Table 5 presents the average levels of enacted, anticipated, and internalized stigma experienced by participants, and bivariate associations between stigma measures and IPV. Enacted sex work stigma in the forms of social discrimination (OR: 3.84, 95% CI: 1.44, 10.25), law enforcement discrimination (OR: 2.75. 95% CI: 1.65, 15.60), and workplace discrimination (job loss) (OR: 3.38, 95% CI: 1.35, 8.46) were associated with increased odds of experiencing IPV. Enacted HIV stigma in the form of workplace discrimination (job loss) was associated with increased odds of IPV (OR: 3.81, 95% CI: 1.77, 8.21). Anticipated stigma in the form of fear that your family could exclude you from family activities if your HIV status were known was also positively associated with IPV (OR: 1.43, 95% CI: 1.00, 2.06).

**Table 5.** Levels of Lifetime Enacted, Anticipated, and Internalized Stigma Experienced and Bivariate Associations With IPV.

	Mean (SD) or percentage (frequency)	OR	95% Wald confidence limits	Parameter est. *p*-value
*Enacted sex work stigma*				
Social discrimination	0.21 (0.33)	3.84	1.44, 10.25	**.007**
Health services discrimination	0.06 (0.17)	2.75	0.45, 16.97	.28
Law enforcement discrimination	0.11 (0.31)	5.07	1.65, 15.60	**.005**
Workplace discrimination (job loss)	10.98% (29)	3.38	1.35, 8.46	**.009**
*Enacted HIV stigma*				
Social discrimination	0.23 (0.33)	2.11	0.76, 5.86	.15
Health services discrimination	0.14 (0.26)	1.96	0.55, 7.02	.30
Workplace discrimination (job loss)	22.35% (59)	3.81	1.77, 8.21	**<.001**
*Anticipated HIV stigma*				
Fear of threat of violence	2.92 (0.92)	1.27	0.83, 1.96	.27
Fear of losing friends' respect	3.06 (0.85)	1.12	0.71, 1.76	.63
Fear of colleagues taking clients	2.27 (1.22)	1.14	0.84, 1.54	.41
Fear of partner abandonment	2.27 (1.20)	0.87	0.63, 1.19	.37
Fear of family exclusion	2.32 (1.03)	1.43	1.00, 2.06	**.05**
*Internalized stigma*				
Sex work	2.21 (0.68)	1.40	0.81, 2.41	.23
HIV	2.22 (0.69)	1.56	0.92, 2.64	.10

Note: Associations with *p*-value ≾ .05 considered significant and highlighted in bold.

### Multivariable Analyses

In the final model (Table 6), the association between workplace HIV discrimination (job loss) and IPV was significant, adjusting for all other forms of stigma and controls. Those who had experienced workplace HIV discrimination (job loss) had 5.60-times the odds of IPV compared to those who had not (95% CI: 1.94, 16.18). The adjusted association between anticipated HIV stigma in the form of fear that your family could exclude you from family activities if your HIV status were known and IPV was also significant (aOR: 1.78, 95% CI: 1.12, 2.82). No other measures of stigma were significantly associated with IPV. 

**Table 6.** Adjusted Odds of IPV (Last 6 Months) by Experiences of Stigma Among Female Sex Workers Living With HIV in Santo Domingo (*n* = 266).

	AOR	95% Wald confidence limits		Parameter estimate *p*-value
*Enacted sex work stigma*				
Social discrimination	2.04	0.45	9.21	.35
Health services discrimination	1.29	0.08	19.77	.86
Workplace discrimination (job loss)	1.62	0.45	5.88	.46
Law enforcement discrimination	3.06	0.69	13.53	.14
*Enacted HIV stigma*				
Social discrimination	0.39	0.08	1.85	.23
Health services discrimination	0.75	0.12	4.58	.76
Workplace discrimination (job loss)	5.60	1.94	16.18	**.002**
*Anticipated HIV stigma*				
Fear of threat of violence	1.33	0.76	2.35	.32
Fear of losing friends' respect	0.70	0.35	1.39	.31
Fear of colleagues taking clients	0.95	0.64	1.42	.82
Fear of partner abandonment	0.82	0.55	1.23	.33
Fear of family exclusion	1.78	1.12	2.82	**.02**
*Internalized stigma*				
Sex work	1.14	0.52	2.48	.74
HIV	1.59	0.65	3.88	.31
*Controls*				
Educational attainment	0.59	0.22	1.56	.29
Age	0.94	0.89	0.99	**.02**
Civil status:				
Cohabitating steady partner (vs. no partner)	5.32	1.01	28.02	**.05**
Non-cohabitating steady partner (vs. no partner)	1.48	0.25	8.78	.67
Number of sexual partners/month	1.01	0.97	1.06	.64

*Notes*. AOR = adjusted odds ratio; IPV = intimate partner violence; Associations with *p*-value ≾ .05 considered significant and highlighted in bold.

In sensitivity analysis, we ran three models containing all measures of one stigma mechanism only (enacted, anticipated, or internalized), adjusting for controls. We found the significance and magnitude of relationships in these separate adjusted models and in the final adjusted model containing all stigma measures to be equivalent. Multicollinearity amongst final model explanatory variables was not problematic per Pearson correlation coefficients (see Table 7) and variance inflation factors.

**Table 7.** Stigma Variables Pearson Correlation Coefficients Matrix.

		Enacted sex work				Enacted HIV			Anticipated HIV					Internalized	
		*Social*	*Health*	*Work*	*Law*	*Social*	*Health*	*Work*	*Violence*	*Friends*	*Col.*	*Partner*	*Family*	*SW*	*HIV*
Enacted sex work	Social	1.00													
												
	Health	0.35	1.00												
		**<.001**													
	Work	0.37	0.30	1.00											
		**<.001**	**<.001**												
	Law	0.30	0.17	0.20	1.00										
		**<.001**	**0.01**	**<.001**											
Enacted HIV	Social	0.54	0.24	0.28	0.17	1.00									
		**<.001**	**<.001**	**<.001**	**<.001**										
	Health	0.22	0.49	0.15	0.20	0.31	1.00								
		**<.001**	**<.001**	**0.02**	**<.001**	**<.001**									
	Work	0.20	0.16	0.42	0.13	0.34	0.25	1.00							
		**.001**	**0.01**	**<.001**	**0.04**	**<.001**	**<.001**								
Anticipated HIV	Violence	0.08	0.14	0.05	0.02	0.00	0.05	−0.02	1.00						
		0.19	**0.02**	0.37	0.76	0.98	0.42	0.80							
	Friends	0.13	0.18	0.12	0.05	0.04	0.03	−0.08	0.57	1.00					
		**0.03**	**<.001**	**0.05**	0.46	0.47	0.61	0.20	**<.001**						
	Colleagues	0.25	0.18	0.14	0.27	0.14	0.18	0.04	0.19	0.26	1.00				
		**<.001**	**<.001**	**0.02**	**<.001**	**0.03**	**<.001**	0.47	**<.001**	**<.001**					
	Partner	0.03	0.00	0.00	−0.08	−0.06	−0.07	−0.06	0.04	0.11	0.05	1.00			
		0.63	0.98	0.99	0.19	0.36	0.26	0.32	0.49	0.08	0.40				
	Family	0.14	0.05	−0.05	0.07	0.13	0.08	−0.08	0.15	0.25	0.12	0.21	1.00		
		**0.02**	0.41	0.42	0.26	**0.03**	0.18	0.21	**0.02**	**<.001**	0.06	**<.001**			
Internalized	SW	0.14	−0.01	0.08	0.04	0.12	0.06	−0.03	0.10	0.23	0.15	0.14	0.20	1.00	
		**0.02**	0.83	0.18	0.50	**0.04**	0.37	0.57	0.09	**<.001**	**0.01**	**0.02**	**<.001**		
	HIV	0.21	0.09	0.08	0.11	0.22	0.05	−0.04	0.30	0.38	0.24	0.19	0.27	0.63	1.00
		**<.001**	0.13	0.20	0.07	**<.001**	0.37	0.51	**<.001**	**<.001**	**<.001**	**<.001**	**<.001**	**<.001**	

Note: Associations with *p*-value ≾ .05 considered significant and highlighted in bold.

## Discussion

We found that enacted HIV stigma (job loss due to HIV) and anticipated HIV stigma (fear of family exclusion due to HIV) were associated with experiencing IPV victimization among FSWs living with HIV. These findings illustrate the economic and social precarity FSWs living with HIV experience due to stigma in combination with other factors. Per Judith Butler, precarity is “the politically induced condition in which certain populations suffer from failing social and economic networks . . . becoming differentially exposed to injury, violence, and death” (Butler, 2009, p. 25). Precarity often refers to “uncertain, unpredictable, and risky” conditions of labor (Kalleberg, 2009, p. 2). In addition to detrimental impacts of depleted economic resources and weakened social ties due to stigma on IPV risk, we argue below that the uncertainty and fear of the potential for such losses characterizing a state of economic and social precarity may also account for relationships between stigma and IPV.

A fifth of participants reported ever having lost a job due to HIV, which may capture discrimination experiences in workplaces in- or outside the sex industry that are commonly reported by FSWs and other women living with HIV in the DR. These include FSW colleagues in direct establishments outing others' HIV status or physically assaulting them due to their status, leading to loss of employment or clients (Brennan, 2004; Zulliger et al., 2017), and HIV testing by employers of hotels, restaurants, factories, and domestic services, leading to firing and exclusion from hiring (Barrington et al., 2017; Human Rights Watch, 2004; Rael et al., 2016; Zulliger et al., 2017). Among women who are not already involved in sex work, losing a job due to HIV discrimination leads some to enter the sex industry (Human Rights Watch, 2004). Possibly reflecting this, 19% of participants in the parent study reported first becoming involved in sex work following their HIV diagnosis. Job loss due to HIV discrimination may increase the odds of IPV by reducing their available economic resources (Caridad Bueno & Henderson, 2017; Deere & Doss, 2006; Friedemann-Sánchez, 2006; Safa, 1995). Our finding is consistent with a study that found unemployed ever married women in the DR had nearly twice the predicted probability of experiencing IPV compared to their employed counterparts, with the strongest effect among asset-poor urban women (Caridad Bueno & Henderson, 2017). Women of low education levels in the DR generally face great difficulty in supporting themselves alone due to low wages, high costs of living and dependent care, and inadequate social protections (Brennan, 2004; Cabezas, 2009; Caridad Bueno, 2015; Gregory, 2007; Lavigne & Vargas, 2013; Safa, 1995). Initiating or maintaining relationships with male partners to share these burdens—which, for PLHIV, include costs related to life-saving HIV care and treatment—may be a necessary or preferable arrangement, even when relationships are abusive (Barrington et al., 2012; Brennan, 2004; Gregory, 2007; Safa, 1995; Zulliger et al., 2017).

Depleted economic resources may also influence the risk of IPV in this population by causing financial stress that degrades intimate partner relationship well-being. Social stress theory (Pearlin, 1989) indicates that poverty imposes strain on individuals that results in conflict with others. The strain of poverty affects aspects of intimate partner relationship well-being that correlate with IPV risk, including relationship satisfaction and levels of conflict, in empirical studies with mainly non-sex worker populations (e.g., Capaldi et al., 2012; Jackson et al., 2009; Neff & Karney, 2017; Rosenblatt & Keller, 1983; Straus, 1990). Financial strain may undercut relationship well-being by increasing opportunities for conflict, degrading communication, increasing the likelihood partners’ problematic personality traits are expressed, and diminishing resources for nurturing shared experiences (Bodenmann et al., 2007; Neff & Karney, 2017). Strain due to the female partner's job loss may be especially pronounced if she had previously contributed substantially to household economic resources, a common scenario within women's intimate partner relationships in the DR where men's economic opportunities are also highly constrained (Barrington et al., 2012; Safa, 1995). Stress, conflict, and IPV in intimate partner relationships may, alternatively, arise in response to a female partner's new HIV diagnosis alone (Arregui, 2007); this could account for the association between job loss due to HIV and IPV, given that many people receive their diagnosis through employer testing that leads to job loss. Future research should explore this possibility, as well as indirect effects of workplace HIV discrimination on IPV via relationship well-being.

Adverse psychological and behavioral effects are another pathway through which HIV-related job loss may relate to IPV. Job loss and denial of work opportunities can cause depression and undermine self-esteem and feelings of autonomy among PLHIV (Barrington et al., 2017; Human Rights Watch, 2004). Depression and low self-esteem may, in turn, increase the risk of experiencing IPV by depleting resolve or perceived capacity to leave abusive relationships (Bacchus et al., 2018; Hong et al., 2013; Logie et al., 2019; Rock et al., 2017). Negative emotional states can also spur affect regulation behaviors like alcohol consumption, which may increase IPV victimization risk among FSWs (Cooper et al., 1995; Heylen et al., 2019; Hong et al., 2013). Minority stress theory-based studies—which examine the disproportionate disease burden borne by marginalized groups due to the social stress of stigma (Meyer, 2003)—show that negative psychological responses to stigma, such as depression and rumination, can degrade intimate partner relationship wellbeing, which can in turn increase the risk of IPV (Doyle & Molix, 2015). The mediating role of mental health in the stigma/IPV relationship is another important area of future research.

The relationship we found between fear of being excluded from family activities due to HIV and IPV could reflect additional ways that stigma may increase IPV vulnerability via undermining effects on social relationships. Women living with HIV and FSWs have described how such anticipated stigma prevents them from disclosing their HIV and/or sex work to family and friends, leading to feelings of detachment and social isolation (Benoit et al., 2018; Carrasco et al., 2017; Logie et al., 2011; Rael et al., 2016; Sandelowski et al., 2004). Weakened relationships with family and friends may diminish emotional and instrumental social support for contending with IPV, such as encouragement, self-esteem bolstering, and help overcoming practical difficulties of terminating abusive relationships (e.g., a place to stay, cash; Caridad Bueno & Henderson, 2017; Go et al., 2011; Rock et al., 2017). FSWs in a study in China reporting one or fewer social network members to go to for financial support in crisis situations had 2.5 times the odds of IPV compared to FSWs reporting more (Hail-Jares et al., 2015). Fear of stigmatization by family and friends due to one's HIV status may be particularly potent for women who are sex workers, given the intersecting, mutually reinforcing nature of HIV stigma and sex work stigma—PLHIV who acquire or are assumed to have acquired HIV via sex work perceive their association with sex work to increase the level of HIV stigma they experience (Katz et al., 2013; Lawless et al., 1996; Sandelowski et al., 2004). Future studies that include sex workers and non-sex workers may shed more light on the influence of sex worker status and sex work stigma on the associations between HIV stigma and IPV that we found.

Finally, HIV stigma may influence IPV risk among FSWs living with HIV not only by spurring economic loss, but also by creating a precarious state of uncertainty and fear of such loss in a context where protection is provided neither by anti-workplace HIV discrimination law nor sex worker labor rights (Barrington et al., 2017; Brennan, 2004; Kempadoo & Doezema, 1998; Lavigne & Vargas, 2013; Rael et al., 2016). When economic disaster due to HIV stigma does strike, the State-provisioned safety net and help from social networks cannot be relied upon, given the inadequacy of social protections and widespread poverty. Economic precarity associated with HIV stigma and these additional socioeconomic conditions may intensify the importance for FSWs living with HIV of securing and maintaining intimate partner relationships as a safeguard for economic shocks, and thus impede avoidance or rejection of abusive but economically supportive relationships. Social precarity due to the perpetual threat of rupture of social ties with family and isolation because of one's HIV status may also increase the difficulty of ending relationships with intimate partners that are abusive but simultaneously constitute sites of love, companionship, and support, as can be the complicated reality in violent intimate partner relationships (Willan et al., 2019).

Since only HIV stigma variables were associated with IPV, our results could be understood to indicate that HIV stigma is more impactful than sex work stigma among these FSWs living with HIV. It is possible that, in this context, HIV stigma is experienced with more frequency, magnitude, or severity—attributes not captured by the binary, lifetime stigma survey items used—leading to stronger relationships with IPV. Additional enacted stigma indicators and/or indicators with more response options capturing greater variability could help to assess why only effects of HIV stigma were significant. It is also plausible that effects of HIV stigma and not sex work stigma were detected due to greater validity of the HIV measures, given that their design was based on a much larger measurement literature. Future research on sex work stigma's effects should consider employing the recent validated sex work stigma scale developed by Kerrigan et al. (2021).

### Intervention Implications

Our findings of positive relationships between workplace HIV discrimination and anticipated social HIV discrimination and IPV suggest that interventions that reduce or buffer effects of these forms of stigma could have positive impacts on the IPV risk of FSWs living with HIV in this context. For instance, anti-discrimination legislation to protect FSWs, PLHIV, and other stigmatized populations in a number of spaces (e.g., workplaces, public spaces, etc.), currently under consideration in the Dominican Republic (Amnesty International, 2019; Parliamentarians for Global Action, 2016), could deter employers’ discriminatory behavior and reduce anticipated social discrimination by promoting social norms of non-discrimination (Loutfy et al., 2015; Stangl et al., 2013). It would be imperative that adequate oversight and accountability mechanisms accompany the law, as demonstrated by the pervasiveness of workplace HIV discrimination despite anti-HIV discrimination legislation. The impotence of this legislation has been attributed to failures of governmental enforcement mechanisms and workers’ lack of means of redress (International Labour Organization, 2014). HIV-focused FSW community empowerment interventions have been shown to reduce stigmatizing attitudes and discriminatory behavior toward sex workers and PLHIV among the general public (Beattie et al., 2010; Biradavolu et al., 2009; Blankenship et al., 2010; Kerrigan et al., 2015), and could thus reduce this population's experiences of enacted and anticipated HIV stigma within their social networks. Individual counseling, which FSWs living with HIV in this context have described as helpful for rejecting stigma, building self-esteem, and improving mental health, may also support them in extricating from abusive relationships (Kerrigan et al., 2018; Rock et al., 2017). Research to evaluate the impact of these interventions on IPV risk among FSWs is warranted.

### Limitations

Limitations of this study include only administering IPV questions to a subset of participants. Not assessing psychological violence may have led to underestimation of IPV prevalence, and the cross-sectional study design limits causal interpretations of findings. The measure of job loss due to HIV discrimination used did not identify whether the discrimination occurred in- or outside the sex industry, information which will be needed for determining programmatic and policy responses appropriate for discrimination within different workplaces—future research should use a more precise measure. Fewer forms of stigma were associated with IPV than expected. Given that the enacted stigma variables were lifetime measures and thus may have captured experiences that occurred long ago, it is plausible that their effects on IPV were not detected because they had decayed by the time of the survey. Future studies should consider including measures of both recent and lifetime stigma experiences.

## Conclusion

This study expands the limited literature by describing levels of enacted, anticipated, and internalized HIV and sex work stigma experienced by FSWs living with HIV and identifying correlates of IPV. Enacted and anticipated HIV stigma may increase IPV vulnerability among FSWs living with HIV by undercutting their economic resources and access to social support, and creating an ever-present threat of such losses. Policies and programs such as community empowerment interventions, anti-discrimination legislation, and individual counseling -- which can reduce the level of HIV and sex work stigma with which FSWs living with HIV contend, and bolster their resilience against it--hold promise for decreasing their vulnerability to IPV. To further advance understanding of pathways through which stigma relates to IPV for the development of targeted interventions, longitudinal research should assess indirect effects via social support, mental health, and relationship wellbeing.
